# The Role of the Carboxyl Terminus Helix C-D Linker in Regulating KCNQ3 K^+^ Current Amplitudes by Controlling Channel Trafficking

**DOI:** 10.1371/journal.pone.0145367

**Published:** 2015-12-21

**Authors:** Frank S. Choveau, Jie Zhang, Sonya M. Bierbower, Ramaswamy Sharma, Mark S. Shapiro

**Affiliations:** 1 Department of Physiology, University of Texas Health Science Center at San Antonio, San Antonio, Texas, United States of America; 2 Department of Cellular and Structural Biology, University of Texas Health Science Center at San Antonio, San Antonio, Texas, United States of America; Sackler Medical School, Tel Aviv University, ISRAEL

## Abstract

In the central and peripheral nervous system, the assembly of KCNQ3 with KCNQ2 as mostly heteromers, but also homomers, underlies “M-type” currents, a slowly-activating voltage-gated K^+^ current that plays a dominant role in neuronal excitability. KCNQ3 homomers yield much smaller currents compared to KCNQ2 or KCNQ4 homomers and KCNQ2/3 heteromers. This smaller current has been suggested to result either from divergent channel surface expression or from a pore that is more unstable in KCNQ3. Channel surface expression has been shown to be governed by the distal part of the C-terminus in which helices C and D are critical for channel trafficking and assembly. A sequence alignment of this region in KCNQ channels shows that KCNQ3 possesses a longer linker between helix C and D compared to the other KCNQ subunits. Here, we investigate the role of the extra residues of this linker on KCNQ channel expression. Deletion of these residues increased KCNQ3 current amplitudes. Total internal reflection fluorescence imaging and plasma membrane protein assays suggest that the increase in current is due to a higher surface expression of the channels. Conversely, introduction of the extra residues into the linker between helices C and D of KCNQ4 reduced current amplitudes by decreasing the number of KCNQ4 channels at the plasma membrane. Confocal imaging suggests a higher fraction of channels, which possess the extra residues of helix C-D linker, were retained within the endoplasmic reticulum. Such retention does not appear to lead to protein accumulation and activation of the unfolded protein response that regulates protein folding and maintains endoplasmic reticulum homeostasis. Taken together, we conclude that extra helix C-D linker residues play a role in KCNQ3 current amplitudes by controlling the exit of the channel from the endoplasmic reticulum.

## Introduction

Voltage-gated KCNQ channels are ubiquitously expressed in human tissues where they play critical roles in the heart, ear, nerves, smooth muscle, and epithelial tissue [1]. In the mammalian brain, KCNQ2 and KCNQ3 regulate the excitability in the central and peripheral nervous system. These channels share a similar topology with other KCNQ channels consisting of tetramers, with each subunit containing six transmembrane domains (S1-S6) and the cytoplasmic amino and carboxyl termini. However, KCNQ2 and KCNQ3 homomers yield currents 10 fold-smaller than those of KCNQ4 homomers, or KCNQ2/3 heteromers [2,3]. Three different regions have been suggested to underlie the divergent expression of KCNQ channels. The first is the N-terminus, which is required for channel trafficking [4] and open probability [5]. The second is the pore region in which networks of interactions between the pore helix and the selectivity filter [6,7] and between the pore helix and the S6 domain [8,9] have been shown to control the channel conductive pathway. The third is the C-terminus, which has been suggested to be involved in channel assembly and surface expression [5,10–12].

Here, we further investigated the role of the C-terminus in KCNQ current expression, focusing on the distal part of the C-terminus, in which helices C and D are required for channel assembly and trafficking [5,11,12]. A number of lines of evidence support the idea that the divergent macroscopic currents of KCNQ channels results at least in part from differential surface expression of channels governed by the distal part of the C-terminus. Thus, replacement of the distal part of the C-terminus of KCNQ1 by that of KCNQ3 increases channel current amplitudes [10]. Moreover, substitution of the D-helix within the distal part of the C-terminus of KCNQ2 by that of KCNQ1 leads to an increase in current amplitudes and, correspondingly, the number of channels at the plasma membrane [11]. However, other studies do not show such a correlation between surface expression and current amplitude. Indeed, the F622L mutation and the D631S/G633E double mutations in the D-helix of KCNQ4, which have been suggested to promote channel tetramerization, did not modify the current amplitude. Consistent with this, we also showed that the C643A mutation at the end of the D-helix in KCNQ4 impaired channel tetramerization but not macroscopic current amplitude [9]. Taken together, these results suggest the distal part of the C-terminus to govern channel assembly and surface expression, but the involvement of this domain in channel current amplitude remains controversial.

**Fig 1:**
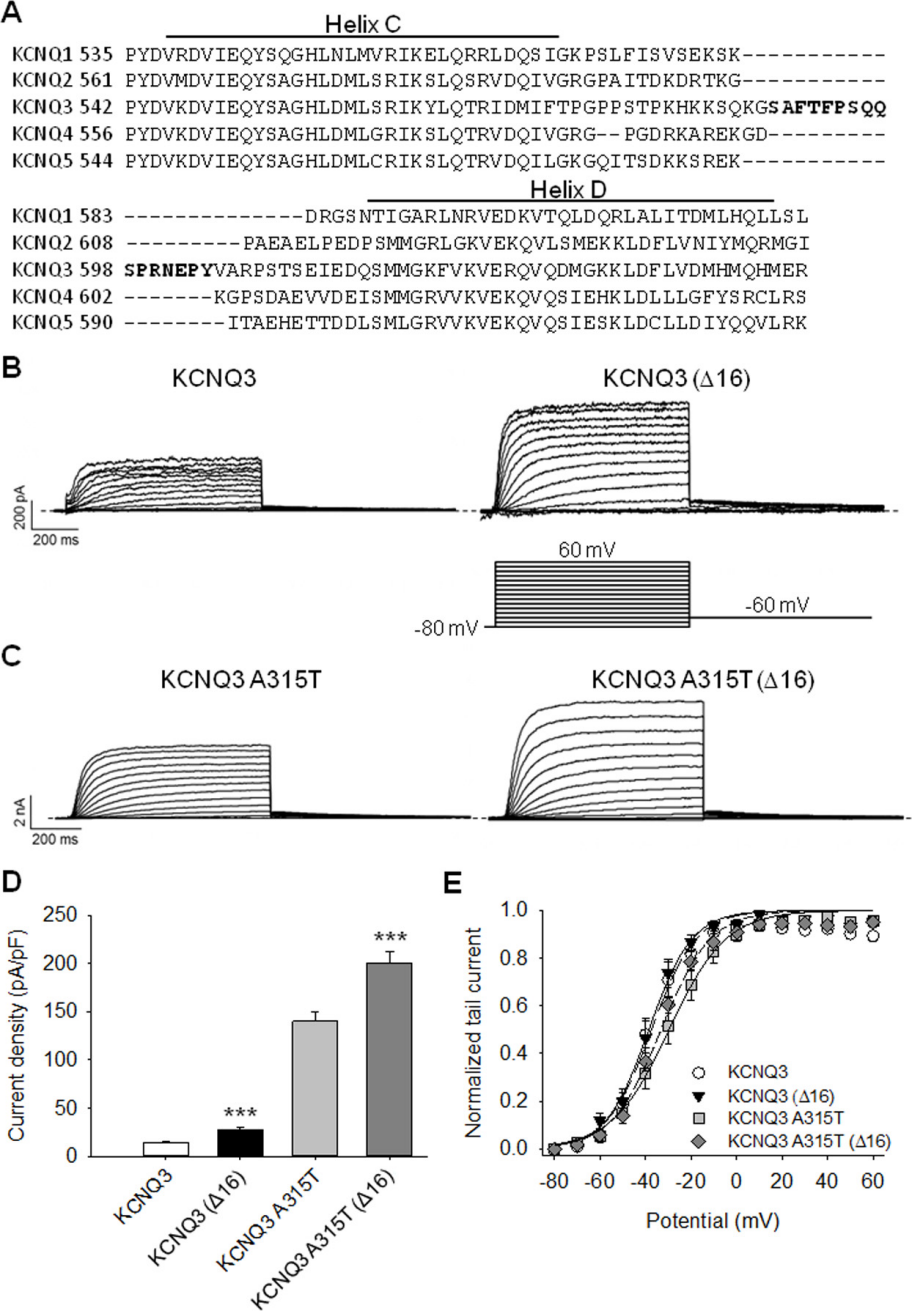
Effects of the deletion of the extra helix C-D linker residues on homomeric KCNQ3 and KCNQ3 A315T currents. (A) Sequence alignment of the helix C-D linker region investigated in this study. Shown from the helix C to the helix D of the part of the C-terminus is an alignment of KCNQ1-5 channels. The residues deleted or inserted in KCNQ3 or KCNQ4 are indicated in bold. The alignment was performed using the program Vector NTI, which takes into account secondary structure in the alignment algorithm. (B and C) Representative perforated patch-clamp recordings from cells transfected with WT KCNQ3, KCNQ3 (Δ16), KCNQ3 A315T or KCNQ3 A315T (Δ16). As shown in the inset, cells were held at -80 mV and voltage steps were applied from -80 to 60 mV in 10 mV increments. (D) Bars show summarized current densities at 60 mV for the indicated channels (n = 6–9; **p<0.01, ***p<0.001). (E) Voltage dependence of activation measured from the normalized amplitudes of the tail currents at -60 mV, plotted as a function of test potential (n = 6–9).

Sequence alignment of the distal part of the C-terminus of KCNQ1-5 indicates that the linker between helices C and D (helix C-D linker) of KCNQ3 contains additional residues compared to that of the other KCNQ subtypes (Fig 1A). The fact that KCNQ3 channels yield a smaller macroscopic current and possess a significantly divergent helix C-D linker compared to other KCNQ subtypes, suggest that the helix C-D linker may play a role in KCNQ3 current amplitudes. To test this prediction, we studied the effect of the deletion of these 16 residues, absent in other KCNQ subtypes (Fig 1A, *bold*), on KCNQ3 channels. Our results show that the deletion of these residues increased homomeric KCNQ3 currents. Biochemical assays, total internal reflection fluorescence (TIRF) microscopy and plasma membrane protein blots, showed that the increase of current amplitudes arises from a higher number of channels at the plasma membrane. Finally, we performed confocal imaging experiments to determine whether the extra 16 helix C-D linker residues play a role in channel trafficking. The proportion of KCNQ3 mutant channels retained in the endoplasmic reticulum (ER) was lower compared to that of wild-type channels, suggesting that this domain may be involved in the retention of channels in the ER. Consistent with this, introduction of these extra helix C-D linker residues of KCNQ3 into KCNQ4 led to a higher retention of channels within the ER. We conclude that the helix C-D linker is partially responsible for the smaller current amplitude of KCNQ3 channels compared to other KCNQ subtypes by controlling KCNQ3 channel exit from ER.

## Materials and Methods

### cDNA Constructs

Human KCNQ3 and KCNQ4 (Genbank accession number AAC96101and AF105202, respectively) were kindly given to us by Thomas Jentsch (Zentrum fur Molekulare Neurobiologie, Hamburg Germany). For TIRF experiments, plasmids were sub-cloned into pEYFP-N1 vectors (Clontech, Mountainview, CA) using standard techniques. Myc-tagged KCNQ3 and KCNQ4 (wild-type and mutants) were generated by sub-cloning each channel in frame into cytomegalovirus-myc (pCMV) vector (Clontech) after the myc epitope. KCNQ3 (Δ16), KCNQ2 (+16) and KCNQ4 (+16) were made by Genewiz, Inc. (South Plainfield, NJ). For confocal imaging, an EcoRI-SalI fragment containing the 16 helix C-D linker residues were excised from Myc-tagged KCNQ3 and ligated into pEGFP-C1 to make a GFP-tagged fusion protein. This construct will produce the EGFP as a tag to the extra 16 helix C-D linker residues placed on the end. This allows us to follow the migration of these 16 residues by tracking the EGFP tag.

### Cell Culture and Transfection

Chinese hamster ovary (CHO) cells were grown in 100-mm tissue-culture dishes (Falcon, Franklin Lakes, NJ) in DMEM medium with 10% heat-inactivated fetal bovine serum plus 0.1% penicillin/streptomycin in a humidified incubator at 37°C (5% CO_2_) and passaged every 4 days. Cells were discarded after approximately 30 passages. For the total internal reflection fluorescent (TIRF) experiments, CHO cells were first passaged onto 35-mm plastic tissue culture dishes and transfected 24 hours later with FuGENE HD reagent (Promega), according to the manufacturer's instructions. For patch-clamp experiments with wild-type and mutant KCNQ3 channels, the cDNA composition transfected was 1 μg KCNQ3 (wild-type) or mutant and 0.3 μg of EGFP. In the KCNQ2 + KCNQ3 experiments, cells were transfected with a ratio 1:1 of KCNQ2 and KCNQ3 (0.8 μg each) and 0.3 μg EGFP. For KCNQ3 A315T experiments, cells were transfected with 0.7 μg of KCNQ3 A315T or KCNQ3 A315T (Δ16) and 0.3 μg of EGFP. For experiments on wild-type or mutant KCNQ4 channels, cells were transfected with 1 μg of wild-type or mutant KCNQ4 and 0.3 μg of EGFP. The next day, cells were plated onto cover glass chips, and experiments were performed over the following 1 to 2 days. For TIRF experiments, CHO cells were transfected with 2 μg of KCNQ3 WT, KCNQ3 (Δ16), KCNQ4 or KCNQ4 (+16).

### Perforated-Patch Electrophysiology

Pipettes were pulled from borosilicate glass capillaries (1B150F-4, World Precision Instruments) using a Flaming/Brown micropipette puller P-97 (Sutter Instruments), and had resistances of 2–4 MΩ when filled with internal solution and measured in standard bath solution. Membrane current was measured with pipette and membrane capacitance cancellation, sampled at 5 ms and filtered at 500 Hz by means of an EPC9 amplifier and PULSE software (HEKA/Instrutech). In all experiments, the perforated-patch method of recording was used with amphotericin B (600 ng/ml) in the pipette [13]. Amphotericin was prepared as a stock solution as 60 mg/ml in DMSO. In these experiments, the access resistance was typically 7–10 MΩ 5–10 minutes after seal formation. Cells were placed in a 500 μl perfusion chamber through which solution flowed at 1–2 ml/min. Inflow to the chamber was by gravity from several reservoirs, selectable by activation of solenoid valves (Warner Scientific). Bath solution exchange was essentially complete by <30 seconds. Experiments were performed at room temperature.

KCNQ currents were studied by holding the membrane potential at -80 mV, and applying 800 ms depolarizing pulses from -80 mV to 60 mV, every 3 seconds. KCNQ-current amplitude was quantified at 60 mV. To estimate voltage dependence, tail current amplitudes were measured approximately 20 ms after the repolarization at -60 mV, normalized, and plotted as a function of test potential. The data were fit with Boltzmann relations of the form: I/I_max_ = I_max_/{1+exp[(V_1/2_-V)/k]}, where I_max_ is the maximum tail current, V_1/2_ is the voltage that produces half-maximal activation of the conductance, and k is the slope factor. Cell populations were compared using two tailed t-test. Data are given as the mean ± SE.

The external Ringer’s solution used to record KCNQ currents in CHO cells contained (in mM): 160 NaCl, 5 KCl, 2 CaCl_2_, 1 MgCl_2_ and 10 HEPES, pH 7.4 with NaOH. The pipette solution contained (in mM): 160 KCl, 5 MgCl_2_ and 10 HEPES, pH 7.4 with KOH with added amphotericin B (600 ng/ml).

### Total Internal Reflection Fluorescence (TIRF) Microscopy

Fluorescence emission from enhanced yellow fluorescent protein (YFP)-tagged KCNQ3, KCNQ3 (Δ16), KCNQ4 WT and the KCNQ4 mutant (+16) was collected at room temperature using TIRF (also called evanescent field) microscopy. TIRF generates an evanescent field that declines exponentially with increasing distance (~300 nm) of the cell very near the cover glass, thus focusing observations to very near the plasma membrane [14]. All TIRF experiments were performed in the TIRF microscopy core facility housed within the Department of Physiology. Images were not binned or filtered, with pixel size corresponding to a square of 122 × 122 nm.

### Plasma Membrane Extraction and Immunoblotting

Cell membrane protein was collected by using a plasma membrane protein extraction kit (Abcam, ab65400). In brief, cells expressing the channel of interest were washed with cold PBS and suspended in homogenized buffer mix in an ice-cold Dounce homogenizer. Homogenates were centrifuged at 700 × g for 10 minutes at 4°C. The resulting supernatants were collected, and centrifuged at 10,000 × g for 30 minutes at 4°C. The resulting supernatants (cytosol) were collected and the pellets (total cellular membrane protein) were re-suspended in upper and lower phase solution. The lysates were again centrifuged at 1000 × g for 5 minutes, and the pellets (membrane) were collected. The distributions of proteins in the membrane fractions were analyzed by Western blots. Protein was run on 10% SDS-Page gels, and then transferred to a nitrocellulose membrane that was probed with the primary antibody (anti-MYC) (Abcam ab32) overnight. The next day, the nitrocellulose membrane was rinsed 3 times with TBS containing tween-20 and incubated with the secondary antibody, anti-mouse IgG, HRP-linked Antibody (Cell Signaling 7076) for 45 minutes. Blots were developed with enhanced chemiluminescence (Supersignal, Pierce) and exposed on X-ray film (Biomax, Rockville, MD). The amount of protein in the lanes of the blot was estimated by measuring the pixel intensity of their corresponding antibody-labeled bars. To control for differing channel proteins made by the cells, the data were quantified as the ratio of surface/total protein for each individual experiment.

For quantifying ER stress-related proteins, CHO cells were transfected with WT or mutant KCNQ3 or KCNQ4 channels. As positive controls, CHO cells were treated with 1 μg/ml of tunicamycin to induce ER stress. Protein extracted after 48 hours using RIPA buffer. Protein concentration was estimated using a BCA assay kit (Pierce, Rockford, IL). Equal amounts of protein (50 μg) were run on 4–20% SDS-polyacrylamide gradient gels (Life Technologies, Carlsbad, CA) and transferred to PVDF membranes. After blocking with 5% non-fat dry milk, membranes were incubated with the following primary antibodies overnight at 4°C: GRP78, total eIF2α, NF-κB p65 (Cell Signaling Technology, Inc., Danvers, MA), phosphorylated eIF2α (Life Technologies), calreticulin (Abcam, Cambridge, MA) and GAPDH (Santa Cruz Biotechnology, Inc., Dallas, TX). Appropriate secondary antibodies and developing reagents (Western Lightning Plus ECL, Perkin Elmer, Waltham, MA) were used for detection. Triplicate transfections were analyzed by densitometry after normalization to loading control.

### Confocal Imaging

Co-localization experiments were performed using a Nikon sweptfield confocal microscope. CHO cells were transfected with cDNAs coding for EGFP, EGFP (+16aa), EGFP-F (membrane targeted), EYFP-tagged KCNQ channels and pDsRED2-ER, which encodes a red-fluorescence protein targeting the ER, using FuGENE HD reagent (Promega). The next day, cells were plated onto glass-bottom dishes, and experiments performed over the following 1 to 2 days. The fluorescent proteins, EGFP, EYFP, and Ds-red, were excited at 488, 514, and 561 nm respectively. All confocal images were generated in the Core Optical Imaging Facility at UTHSCSA. Images were acquired sequentially using a 525/40 nm emission filter for GFP/YFP and a 607/70 nm emission filter for Ds-red. Co-localization analysis was performed using Image J software with the plug-in to calculate the Manders’ coefficient [15]. Similar experiments were performed with EGFP-tagged KCNQ4 (+16) and pDsRED2-ER.

## Results

### Deletion of the Additional 16 Residues in the Helix C-D Linker Increases Current Amplitudes from KCNQ3 and KCNQ3 A315T

Despite a similar topological structure, KCNQ1-5 channels display differences in current amplitudes. Previous studies have investigated the role of the C-terminus in KCNQ current amplitudes [5,10–12]. According to those studies, the divergence of currents among KCNQ channels is mostly due to differential surface expression which is governed by helices C and D within the distal part of the C-terminus. However, other studies have not observed such an effect of the distal part of the C-terminus on current amplitudes, but have rather implicated the channel pore [6,9]. A sequence alignment of the distal part of the C-terminus of KCNQ channels highlights that the linker between helices C and D in KCNQ3 channels contains a domain of 16 extra residues compared to that of other KCNQ subtypes channels (Fig 1A, *bold*). Previous work from several labs has found that position 315 in KCNQ3 to be critical to determining functional expression [6,7,16]. Indeed heterologous expression of WT KCNQ3 results in minute macroscopic currents [6,7,11] or numbers of channels in single-channel patches [17]. The residue at position 315 is uniquely an alanine in KCNQ3, as opposed to hydrophilic residues in the other subtypes, and substitution of that alanine by a threonine or serine increases macroscopic currents by approximately 30-fold [5,6,16], without changing their maximal open probability or apparent affinity for PIP_2_, making their study as homomers feasible [18]. Thus, in our experiments we included the KCNQ3 A315T mutant (called KCNQ3^T^ in previous papers by us and others) as a background channel, and included making mutations on this background, to ensure that any effects of the C-D helix linker were independent of the residue at position 315.

To determine the role of the extra 16 residues (SAFTFPSQQSPRNEPY), uniquely present in the C-D helix linker of KCNQ3 on current amplitudes, we deleted those residues in WT KCNQ3 and KCNQ3 A315T. Then, we studied the effects of this deletion on KCNQ3 and KCNQ3 A315T currents. We found that this deletion increased the current density by nearly 2-fold relative to WT KCNQ3 from 14.9 ± 1.0 pA/pF (n = 7) to 27.5 ± 2.6 pA/pF (n = 6; p<0.001; Fig 1B and 1D). Similar results were obtained with KCNQ3 A315T channels. Thus, KCNQ3 A315T (Δ16) channels produced larger currents than KCNQ3 A315T channels (Fig 1C). CHO cells transfected with KCNQ3 A315T (Δ16) displayed a current amplitude of 201 ± 11 pA/pF (n = 7), whereas those expressing KCNQ3 A315T displayed a current amplitude of 140 ± 9 pA/pF (n = 9; p<0.001) (Fig 1D). Finally, this deletion did not significantly affect the voltage dependence of activation for KCNQ3 and KCNQ3 A315T channels (Fig 1E). For cells transfected with KCNQ3 or KCNQ3 (Δ16), the half-activation potentials were -36.2 ± 3.1 mV (n = 6) and -37.8 ± 2.7 mV (n = 6), respectively. For cells transfected with KCNQ3 A315T or KCNQ3 A315T (Δ16), the half activation potentials were -29.8 ± 3.3 mV (n = 9) and -33 ± 1.7 mV (n = 7), respectively.

### Differential Surface Protein Expression Explains the Divergent Current Amplitudes between KCNQ3 WT and Mutant Channels

**Fig 2:**
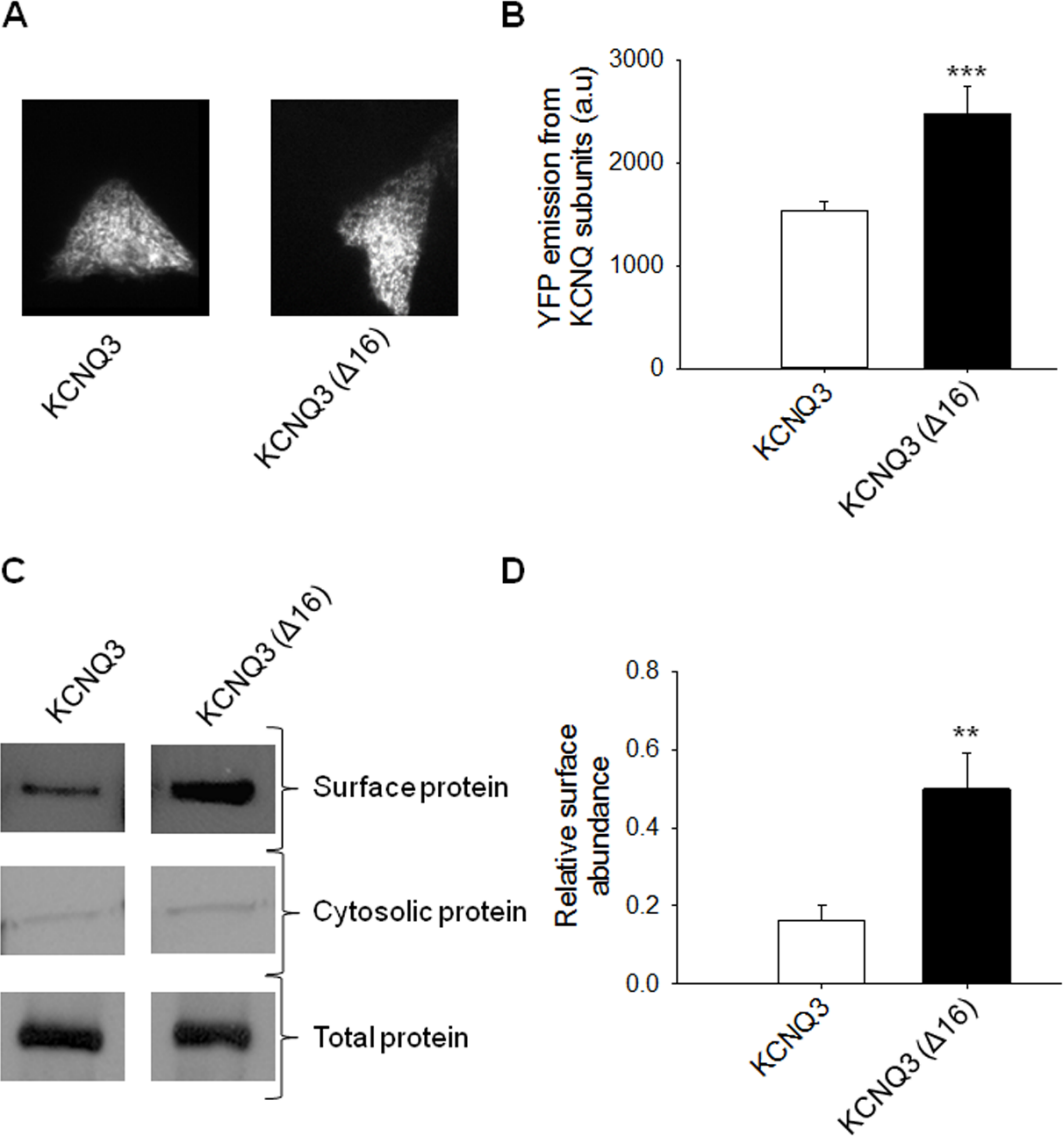
Differential surface protein expression explains the divergent current amplitudes between WT and mutant KCNQ3. (A) Shown are representative fluorescent images from TIRF microscopy of CHO cells expressing YFP-tagged KCNQ3 WT or mutant channels. In all cases, CHO cells were transfected with a total of 2 μg cDNA. (B) Bars show summarized data for each channels (n = 57–63; ***p<0.001). (C) Shown are representative plasma membrane protein assays for the indicated channels. The upper panels show the plasma membrane myc-tagged protein; the middle panels show the cytosolic myc-tagged proteins and the lower panels show the total myc-tagged protein from whole-cell lysates. (D) Bars show summarized plasma membrane protein assays. For each experiment, the ratio of surface/total protein was calculated to estimate relative surface expression. These values were then compared with those of the indicated channels (n = 6; **p<0.01).

To determine whether the increase in the current density of KCNQ3 (Δ16) homomers is due to differences in plasma-membrane expression of the channels, we performed two sets of experiments. First, we measured the plasma-membrane expression for WT KCNQ3 and KCNQ3 (Δ16) channels by examining individual CHO cells using TIRF microscopy. Although, some of our patch-clamp experiments used the A315T mutant as a background, due to its much greater ease of study (see below), we here used the WT channels, since WT KCNQ3 proteins are found to be expressed in the membrane similarly to the A315T mutant. Under TIRF illumination, only fluorophores located within approximately 300 nm of the plasma membrane are excited, and thus only channels expressed at the plasma membrane are illuminated [14]. We constructed EYFP-tagged WT and mutant KCNQ3 and expressed them in CHO cells. Fig 2A shows representative images of cells transfected with EYFP-tagged WT or KCNQ3 (Δ16) channels. The EYFP emission of each channel was quantified as mean pixel intensity over the surface area of the cell. Our results revealed a significant increase in membrane expression of the KCNQ3 mutant (Δ16) (2475 ± 262 arbitrary units (A.U.), n = 57; p<0.001) compared to KCNQ3 WT (1535 ± 91 A.U., n = 63) (Fig 2B). This difference in surface expression is consistent with the increase in the current density of the KCNQ3 (Δ16) compared to KCNQ3 WT channels. We conclude that the differential membrane abundance of channel protein may underlie the differences in macroscopic current amplitudes.

The second assay for plasma-membrane expression was performed using a plasma membrane protein assay to specifically label and quantify KCNQ3 wild-type and mutant (Δ16) channels in the plasma membrane. In these experiments, KCNQ3 WT and mutant (Δ16) subunits were tagged with a myc epitope to their amino terminus, and individually expressed in CHO cells. Fig 2C shows immunoblots of the cell-surface (top row), the cytosol (middle row), and total lysate (bottom row) for each protein. The cytosol fraction is used as an additional control for successful membrane protein purification. We found the cytosolic protein level to be negligible compared to that of membrane protein. These data suggest that the increase in current density is due to an increase in the number of channels at the plasma membrane. Indeed, the surface/total ratio of KCNQ3 WT and KCNQ3 (Δ16) channels were 0.16 ± 0.04 and 0.49 ± 0.09 (n = 6; p<0.01) (Fig 2D), respectively. These results suggest that the extra helix C-D linker residues are involved in channel surface expression.

### Insertion of the Extra Helix C-D Linker Residues of KCNQ3 into that of KCNQ4 Reduces Channel Current Amplitudes and Surface Expression

**Fig 3:**
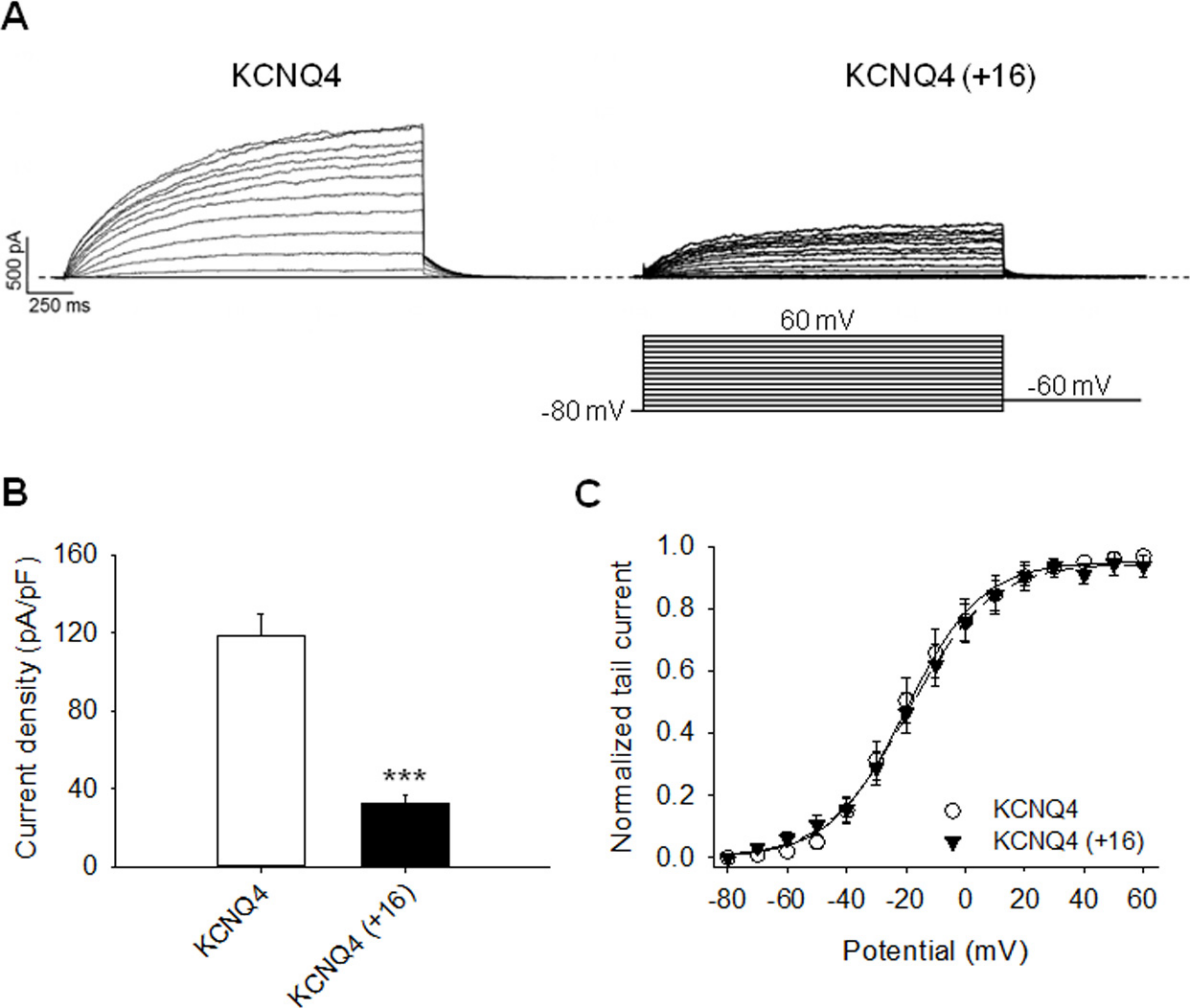
Effects of the extra helix C-D linker residues of KCNQ3 on KCNQ4 currents. (A) Representative perforated patch-clamp recordings from CHO cells transfected with KCNQ4 WT or KCNQ4 (+16). As shown in the inset, cells were held at -80 mV and voltage steps were applied from -80 to 60 mV in 10 mV increments. (B) Bars show summarized current densities at 60 mV for the indicated channels (n = 9–10; ***p<0.001). (C) Voltage dependence of activation measured from the normalized amplitudes of the tail currents at -60 mV, plotted as a function of test potential (n = 7–9).

Our data indicate that the extra helix C-D linker residues play a role in KCNQ3 current amplitudes and surface expression. This suggests that the difference in current between KCNQ3 and other KCNQ channels may partially be due to the presence of the additional 16-residue domain within the helix C-D linker in KCNQ3 channels. To further test this hypothesis, we inserted these extra residues (SAFTFPSQQSPRNEPY) of the helix C-D linker of KCNQ3 into that of KCNQ4, which is known to express robust homomeric currents [6,9,17] and tested the effect insertion of these residues on KCNQ4 currents. Insertion of the 16 helix C-D linker domain of KCNQ3 into that of KCNQ4 decreased macroscopic current density from 118 ± 11 pA/pF (n = 10) to 33 ± 4 pA/pF (n = 9; p<0.001) (Fig 3B). As previously described in KCNQ3 channels, the helix C-D linker does not seem to play a role in the gating machinery since the voltage-dependence of activation was not significantly modified in KCNQ4 (+16) mutant channels (Fig 3C).

**Fig 4:**
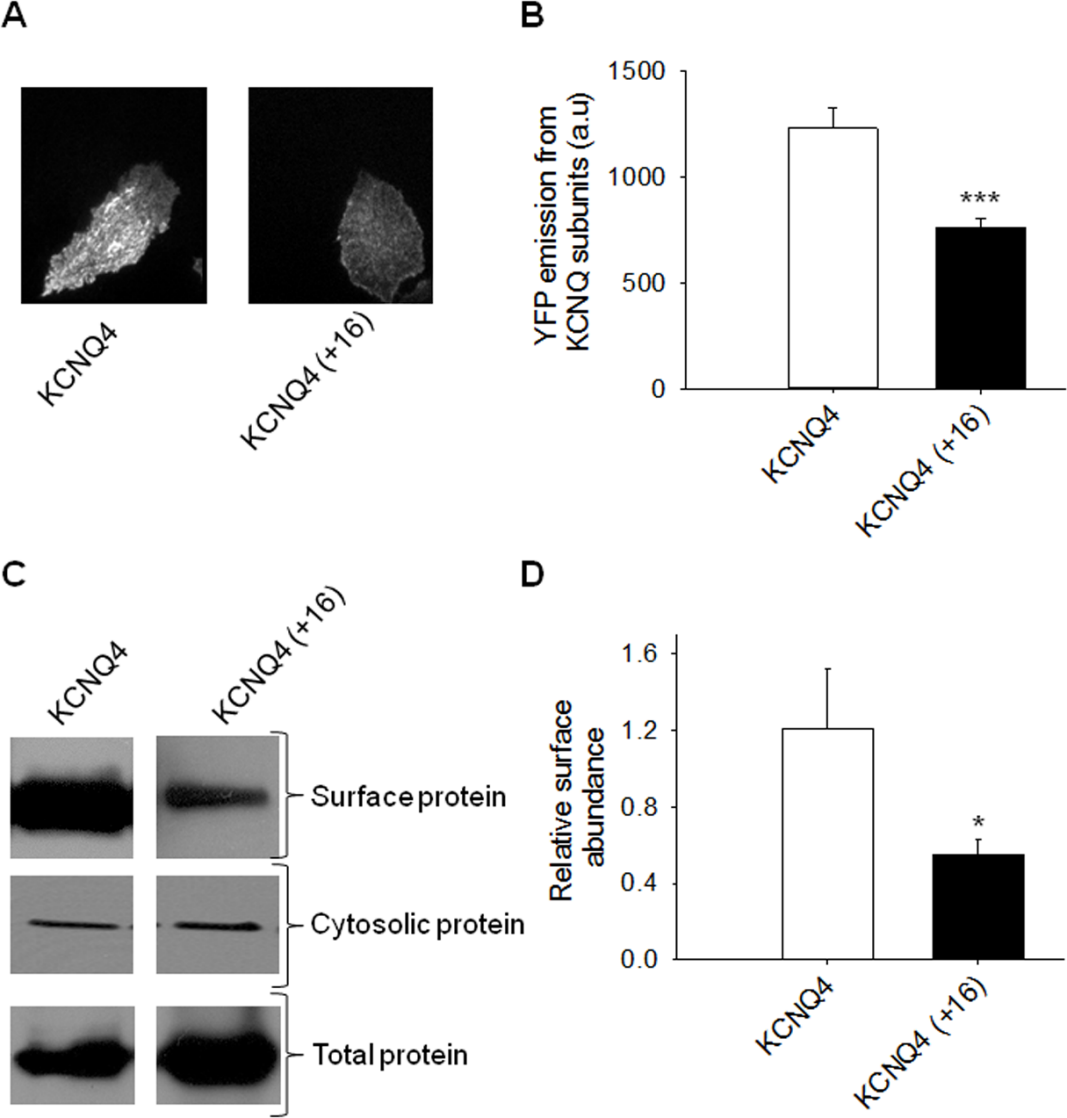
Insertion of the extra helix C-D linker residues of KCNQ3 into that of KCNQ4 reduces channel surface expression. (A) Shown are representative fluorescent images from TIRF microscopy of CHO cells expressing the indicated YFP-tagged KCNQ4 channels. In all cases, CHO cells were transfected with a total of 2 μg cDNA. (B) Bars show summarized data for each channels (n = 110–116; ***p<0.001). (C) Shown are representative plasma membrane protein assays for the indicated channels. The upper panels show the plasma membrane myc-tagged protein, the middle panels show the cytosolic myc-tagged proteins and the lower panels show the total myc-tagged protein from whole-cell lysates. (D) Bars show summarized plasma membrane protein assays. For each experiment, the ratio of surface/total protein was calculated to estimate relative surface expression. These values were then compared with those of the indicated channels (n = 6; *p<0.05).

To determine whether the decrease of current observed in the KCNQ4 (+16) mutant channels is due to a lower surface expression, we performed two sets of experiments. In the first set, we performed total internal reflection fluorescence microscopy (TIRF). To visualize WT and mutant KCNQ4 channels, we tagged each channel with EYFP and expressed them in CHO cells. The fluorescent intensity of EYFP-KCNQ4 (+16) channels under TIRF decreased to 759 ± 41 A.U., compared to WT KCNQ4 (1227 ± 100 A.U., n = 110–116; p<0.001) (Fig 4B). This decrease in surface expression of EYFP-KCNQ4 (+16) channels measured with TIRF was similar to the change in current density measured with whole-cell patch-clamp. Finally, to confirm that the decrease of current in KCNQ4 (+16) channels arises from a lower number of channels at the plasma membrane, we then performed immunoblotting of plasma membrane protein levels in CHO cells expressing myc-tagged WT or KCNQ4 (+16). We found that the protein level of KCNQ4 (+16) channels at the membrane was lower than that of WT KCNQ4 (Fig 4C and 4D). The surface/total ratios of KCNQ4 WT and KCNQ4 (+16) channels were 1.2 ± 0.3 and 0.6 ± 0.1 (n = 6; p<0.05), respectively. These data suggest that the 3- to 4-fold lower density of KCNQ4 (+16) versus KCNQ4 WT channels is mostly due to differences in membrane expression of channel protein.

### The Extra Helix C-D Linker of KCNQ3 Increases the Retention of Channels in the ER

**Fig 5:**
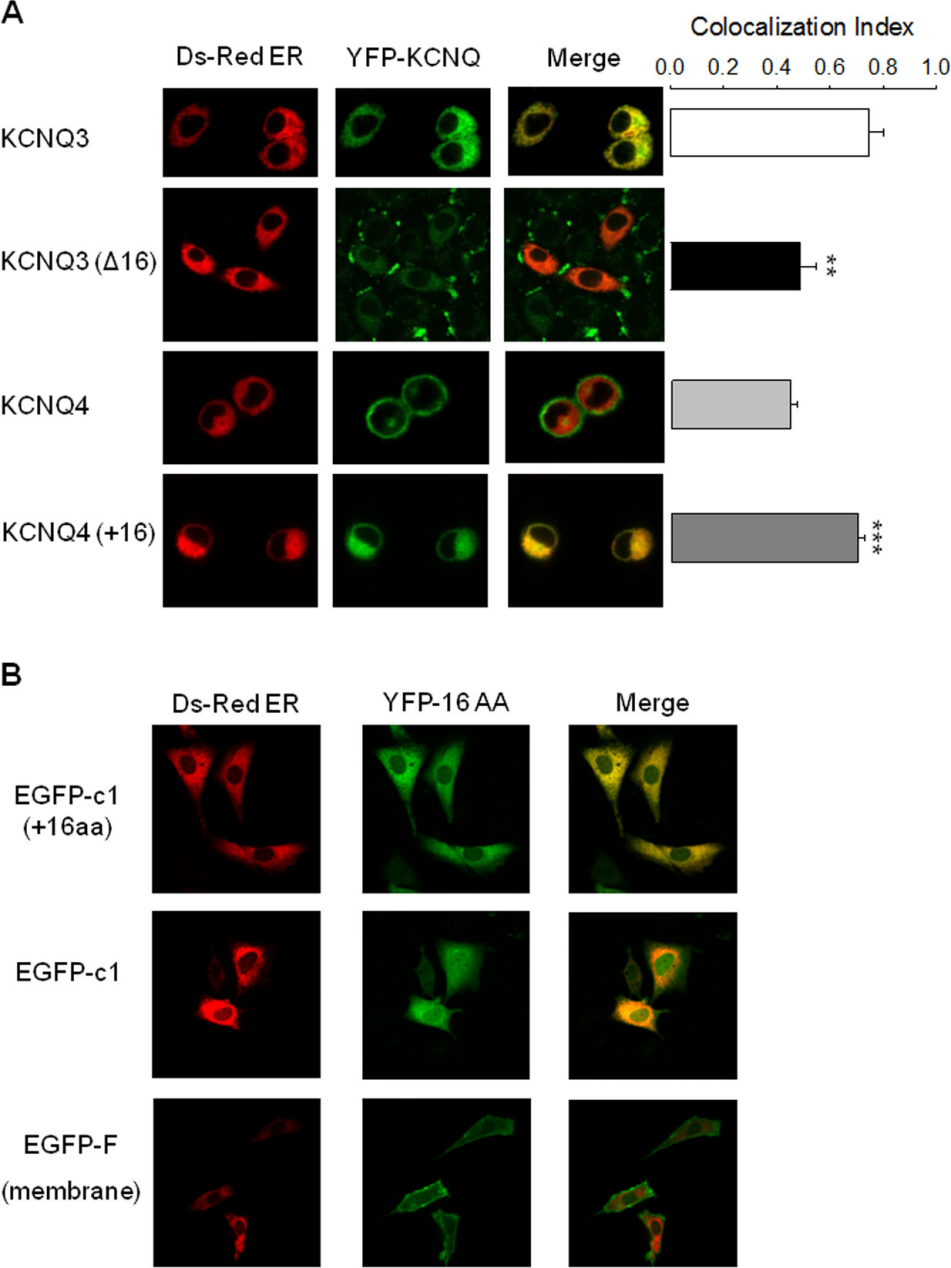
The extra 16 helix C-D linker residues lead to channel retention in the ER. (A) Shown are representative confocal images of CHO cells expressing the indicated YFP-tagged KCNQ channels and an ER marker, pDsRED2-ER. Bars show averaged Manders coefficient for each channel which determines the degree of colocalization between the indicated channel and the ER marker (n = 19–36; **p<0.01, ***p<0.001). (B) Shown are representative confocal images of CHO cells expressing an ER marker, pDsRED2-ER with GFP-tagged 16 additional helix C-D linker residues, EGFP alone or EGFP-F (membrane-localized) (n = 11–15).

Taken together, our results indicate that the extra helix C-D linker 16 residues are partially involved in the smaller current density of KCNQ3 homomers by impairing surface expression. We next analyzed whether these 16 residues linker impair channel trafficking to the plasma membrane. To address this question, we investigated the fraction of channels localized in the ER from cells co-transfected with a DsRed-tagged ER marker together with EYFP-tagged WT KCNQ3 or the KCNQ3 (Δ16) mutant. We first compared the level of co-localization of KCNQ3 WT and KCNQ3 (Δ16) channels with the ER marker. The co-localization analysis revealed that a lower fraction of KCNQ3 (Δ16) channels was retained in the ER compared to WT KCNQ3 (Fig 5A). These results suggest that the extra helix C-D linker may promote the retention of the channel in the ER. If the extra helix C-D linker residues are involved in ER retention of the channel, insertion of these residues into the helix C-D linker of KCNQ4 should increase the fraction of KCNQ4 channels retained in the ER. As shown in Fig 5A, KCNQ4 (+16) channels, which possess the extra C-D linker residues of KCNQ3, are indeed retained more in the ER as compared to WT KCNQ4. These data argue for a role of the extra C-D helix linker residues in ER retention of the channel.

Many studies have investigated signals that prevent channels from exiting the ER and consequently from reaching the plasma membrane. Several types of ER-retention signals have been identified that include di- and tri-basic motifs such as di-lysine (KKxx and KxKxx), di-arginine (RXR), and tri-arginine (RRR) motifs, where X represents any amino acid [19–22]. PL(Y/F) (F/Y) xxN and H/KDEL have also been shown to function as ER retention signals [23–25]. However, none of these motifs are present in the sequence of the additional helix C-D linker residues of KCNQ3 channels (SAFTFPSQQSPRNEPY), suggesting that the higher retention in the ER of the channels, which possess the extra C-D linker residues, may not be due to the presence of a known ER retention signal in the helix C-D linker. To determine whether the helix C-D linker contains an ER retention motif, this extra helix C-D linker residues tagged with a green fluorescent protein, GFP-c1(+16aa), were co-transfected with the ER marker (pDsRED2-ER) in CHO cells to track the localization of the additional helix C-D linker residues. As our controls, CHO cells were co-transfected with (1) EGFP alone in the same vector (EGFP-C1), or (2) EGFP tagged with plasma membrane localization signal (EGFP-F), and the ER marker, DS-Red ER. Confocal imaging showed that EGFP alone is expressed in the cytoplasm, thus partially co-localized with the ER maker; whereas when fused with the known plasma membrane localization signal, EGFP is expressed in the plasma membrane and does not co-localize with the ER marker (Fig 5B). Similarly, when tagged with the 16 additional residues of the helix C-D linker, EGFP expression is strictly resided in ER, demonstrating by the near total co-localization with the ER marker. Thus, these experiments imply that it is these 16 amino acids that bring EGFP to the ER. Although, we acknowledge that EGFP is not a secreted protein that is known to be transported from the ER to the plasma membrane, this additional experiment is consistent with the ER-retention idea suggesting that these 16 amino acids may constitute a, hitherto unknown, ER retention signal.

### ER Retention Does Not Activate Components of the Unfolded Protein Response

**Fig 6:**
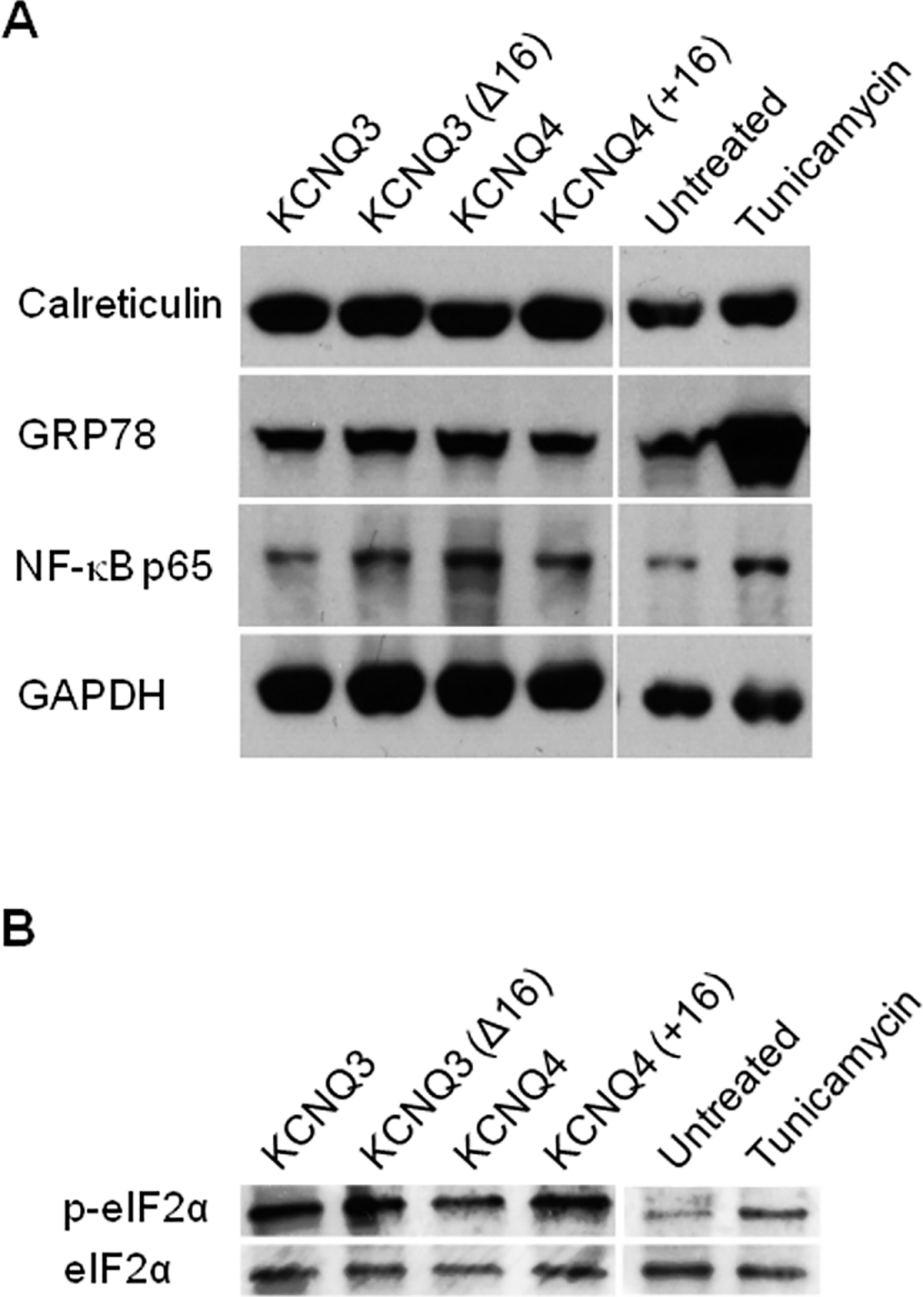
Protein retention does not trigger the unfolded protein response (UPR). CHO cells transiently transfected with the indicated channels were lysed after 48 hours and protein loaded onto SDS-polyacrylamide gels. After transfer, PVDF membranes were probed for molecular chaperone GRP78, NF-κB and for the ER-resident protein, calreticulin (A) and UPR-related proteins such as phospho-eIF2α (B) during the UPR. As controls, CHO cells were treated with 1 ug/ml tunicamycin to induce ER stress. GAPDH and total eIF2α were used as loading controls. Shown are representative immunoblots for the indicated channels (n = 3).

Post ER retention, further folding and secretion of the protein may also be regulated by a stress-based signaling pathway called the Unfolded Protein Response (UPR). Indeed, the UPR has been shown to regulate glutamate receptor export from the ER [26]. The UPR serves to up-regulate protein folding chaperones such as GRP78 while simultaneously attenuating further protein translation by phosphorylating the eukaryotic translation initiation factor 2α [27]. Additionally, the UPR also induces the transcription factor, NF-κB [28] and calreticulin, a chaperone involved in the calnexin-calreticulin cycle for folding of glycoproteins and in ER calcium regulation. To determine if ER retention of KCNQ3 channels resulted in activation of the UPR, we analyzed transfected samples for upregulation of the molecular chaperone, GRP78. As shown in Fig 6A, the presence or absence of the helix C-D linker did not affect BiP expression. We also did not find any significant changes in phosphorylation status of eIF2α (Fig 6B). Similarly, expression levels of NF-κB p65 and the ER resident protein, calreticulin, were unchanged (Fig 6B). As a control, we treated untransfected CHO cells with tunicamycin, a UPR activator to ensure the proper activation of the UPR. These results confirm that the UPR does not play a significant role in modulating KCNQ3 trafficking to the plasma membrane.

### The Extra Helix C-D Linker Residues of KCNQ3 also Decrease KCNQ2 Current Amplitudes Due to a Higher Retention of the Channel in the ER

**Fig 7:**
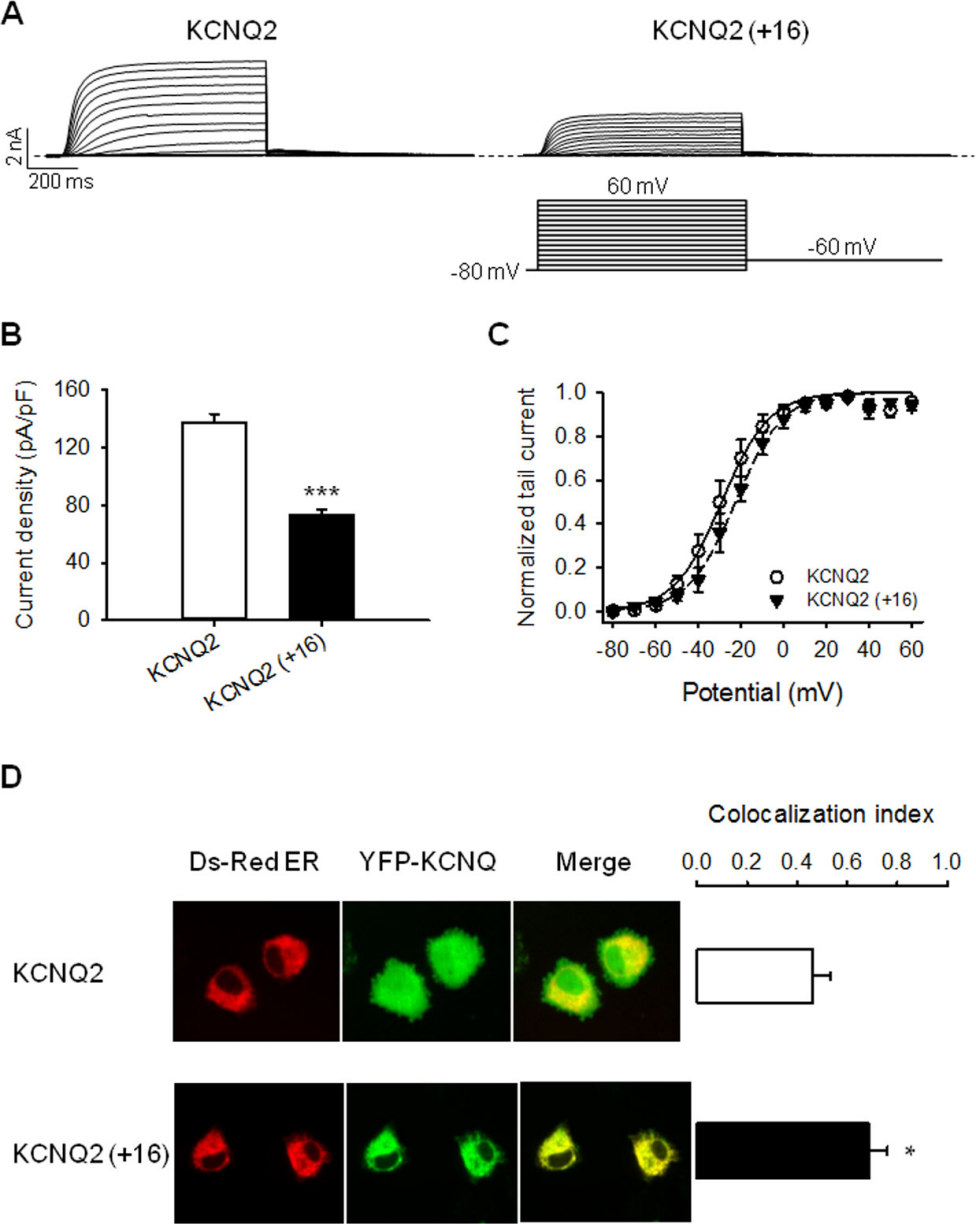
Insertion of the extra helix C-D linker residues of KCNQ3 into that of KCNQ2 channel reduces current amplitudes and increases channel retention in the ER. (A) Representative perforated patch-clamp recordings from cells transfected with KCNQ2 and KCNQ2 (+16). As shown in the inset, cells were held at -80 mV and voltage steps were applied from -80 to 60 mV in 10 mV increments. (B) Bars show summarized current densities at 60 mV for the indicated channels (n = 7–8; ***p<0.001). (C) Voltage dependence of activation measured from the normalized amplitudes of the tail currents at -60 mV, plotted as a function of test potential (n = 5–6). (D) Shown are representative confocal images of CHO cells expressing the indicated EYFP-tagged KCNQ channels and an ER marker, pDs-RED2-ER. Bars show averaged Manders coefficient for each channel which determines the degree of colocalization between the indicated channel and the ER marker (n = 7–10; *p<0.05).

Given the fact that KCNQ2 does not have the additional 16-residue domain within the helix C-D linker, as does KCNQ4, one would expect similar effects of these residues to apply to both channels. To address this question, we inserted these extra residues (SAFTFPSQQSPRNEPY) of the helix C-D linker of KCNQ3 into that of KCNQ2 and studied their effects on currents and KCNQ2 retention in the ER. As for KCNQ4, KCNQ2 (+16) channels yield a smaller current (Fig 7A and 7B) and are retained more in the ER compared to WT KCNQ2, which localizes in the cytoplasm and on the plasma membrane (Fig 7D). These data reinforce the idea that the extra C-D helix linker residues play a role in ER retention of the channels.

### The Additional Helix C-D Linker Residues of KCNQ3 Are Not Involved in Channel Assembly

**Fig 8:**
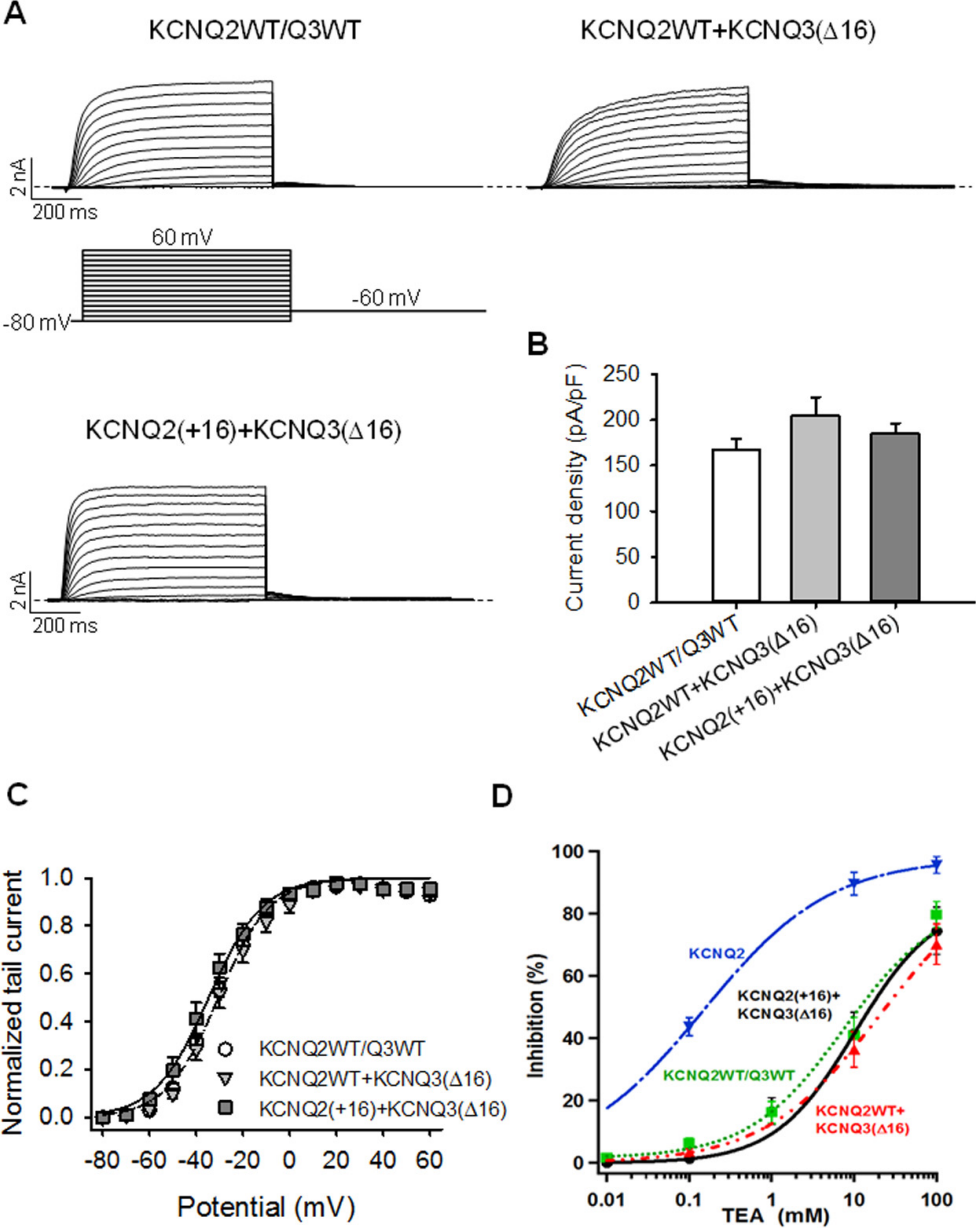
The extra helix C-D linker residues do not affect channel assembly. (A) Representative perforated patch-clamp recordings from cells transfected with KCNQ2+Q3, KCNQ2+Q3 (Δ16) or KCNQ2 (+16) + Q3 (Δ16). As shown in the inset, cells were held at -80 mV and voltage steps were applied from -80 to 60 mV in 10 mV increments. (B) Bars show summarized current densities at 60 mV for the indicated channels (n = 7–11). (C) Voltage dependence of activation measured from the normalized amplitudes of the tail currents at -60 mV, plotted as a function of test potential (n = 7–10). (D) TEA^+^ dose-response curve of each type of channel (n = 4–11). The data were fit by a Hill equation. For all of these, fits the maximal block was constrained to be 100%.

Given the fact that Kv channel tetramerization occurs in the ER [29,30] and for Kv7 (KCNQ) channels is governed by the distal part of the C-terminus (helix C-linker-helix D) [11,12,31,32], we hypothesized that the residues highlighted in this study may induce channel retention in the ER by preventing the formation of stable heteromeric tetramers. To determine whether the helix C-D linker affects such heteromeric channel assembly, we studied the effect of deleting the additional residues of the helix C-D linker on KCNQ2/3 currents (Fig 8). Since, WT KCNQ3 is well known to assemble with KCNQ2 to generate very robust currents, we did not have to use the KCNQ3 A315T mutant in this assay.

We found this the 16-residue deletion to not significantly increase heteromeric KCNQ2/3 currents (Fig 8B). As shown previously in Fig 1, the KCNQ3 mutant (Δ16) displayed larger currents than KCNQ3 WT. In contrast, insertion of these 16 residues of the KCNQ3 helix C-D linker into that of KCNQ2 decreased current amplitudes (Fig 7A and 7B). Interestingly, when both mutant channels were co-expressed, the current produced was similar to that of WT KCNQ2 + WT KCNQ3 (Fig 8A and 8B). These results show that KCNQ2 (+16) and KCNQ3 (Δ16) channels still co-assemble to form heteromers, suggesting that the helix C-D linker is not critical for channel assembly. These data are consistent with the idea that the 16 additional residues of the helix C-D linker constitute an ER retention motif.

In our co-expression experiments, we needed to confirm that both KCNQ2 and KCNQ3 (WT or mutant) subunits are being incorporated in the channel tetramers; i.e., that the changes in current densities observed are not due to omission of KCNQ3 subunits and expression of only KCNQ2 homomers. To verify this, we exploited the very different sensitivity of KCNQ2 homomers and KCNQ2/3 heteromers to block by external tetraethylammonium ions (TEA^+^) [33,34]. TEA was applied over a range of concentrations, and the block of currents quantified using the “classic” M-current protocol [33]. The approximate sensitivity was determined by fitting the data to a single Hill equation, which is sufficient to clearly distinguish between homomeric and heteromeric currents. For KCNQ2 homomers and KCNQ2WT/Q3WT, the IC_50_ were 0.15 ± 0.02 mM and 12.1 ± 2.8 mM (n = 4–11), respectively. The dose-response curve for KCNQ2 (+16) + KCNQ3 (Δ16) was unchanged, with an IC_50_ of 10.3 ± 5.7 mM; whereas that for KCNQ2WT + KCNQ3 (Δ16) was shifted towards higher TEA^+^ concentration, with an IC_50_ of 20.3 ± 6.4 mM (n = 5–7) (Fig 8D). Because channels with two KCNQ2 and two KCNQ3 subunits have a lower TEA^+^ sensitivity than those with three KCNQ2 and one KCNQ3 subunits [33], it is likely that KCNQ2WT + KCNQ3 (Δ16) have one KCNQ2 and three KCNQ3 subunits which would explain its lower TEA^+^ sensitivity compared to KCNQ2WT/Q3WT.

## Discussion

In this study, we further the investigation of the structural mechanisms involved in the diversity of KCNQ1-5 current amplitudes. Many studies have shown that the divergent current amplitudes between KCNQ3 and the other KCNQ channels may be due to differential surface expression of channels [5,10,35]. According to those studies, helices C and D within the distal part of the C-terminus determine current amplitudes by governing channel surface expression and assembly. Thus, Schwake and co-workers showed that KCNQ2/3 heteromers are well expressed at the plasma membrane compared to KCNQ2 and KCNQ3 homomeric channels, due to a “subunit-interaction domain” (SID) that spans the C and D helices [11]. A more recent study highlighted critical salt-bridges between coiled-coiled residues in the D-helix, which were shown to be uniquely lacking in KCNQ3, although site-directed mutagenesis to endow those salt bridges in KCNQ3 did not result in current amplitudes like those for KCNQ4 [12]. Nonetheless the SID and surrounding domains in the distal C-terminus are clearly critical to channel assembly, heteromerization and expression.

We here suggest that the linker between the C and D helices also plays a role in governing current amplitudes by regulating surface expression. Based on a sequence alignment of the helix C-linker-helix D region, we identified 16 residues in the linker between helices C and D in KCNQ3 channels, which are absent in KCNQ4. To investigate the importance of these additional residues on KCNQ3 current amplitude, we studied the effect of the deletion of the extra helix C-D linker residues in KCNQ3 and KCNQ3 (A315T) homomers. This deletion led to an increase in KCNQ3, as well as KCNQ3 (A315T), current amplitudes. Moreover, our TIRF and plasma membrane protein assays showed that this increase results from a higher surface expression compared to WT KCNQ3. Thus, we conclude that the helix C-D linker plays a role in functional expression of KCNQ3 channels. To determine whether the difference in current density between KCNQ3 and other KCNQ channels, such as KCNQ4, is partially due to the extended helix C-D linker of KCNQ3, we studied the effects of the introduction of these residues into the helix C-D linker on KCNQ4 currents. We found that the introduction of this motif into the helix C-D linker of KCNQ4 significantly reduced KCNQ4 currents. Additionally, TIRF microscopy and plasma membrane protein assays revealed that the decrease of currents observed arises from a lower number of channels at the plasma membrane. These results suggest that the extra helix C-D linker residues in KCNQ3 may play a role in the divergence of current between KCNQ3 and KCNQ4 channels.

The results in this manuscript in no way reduce the importance of the profound effect of the residue at the 315 position (located next to the internal TEA binding site of TEA-sensitive Kv channels) on expression of functional channels, for which substitution of the native and unique alanine by a hydrophilic threonine or serine augmented functional expression by nearly 30-fold [5,6,16], without significantly altering the presence of KCNQ3 protein at the plasma membrane [7]. In addition, our work here does not take away the critical nature of the C and D helices in governing channel tetramerization and function (see below). What is clear though, as highlighted in work from the Villarroel lab [5], is that KCNQ channels possess multiple domains involved in assembly, export to the membrane, and functional expression, each playing some varying, yet significant, role. The Lotan lab has also indicated the importance of the amino terminus in functional interactions with the pre-synaptic protein, synataxin, consistent with multiple sites within KCNQ channels to play parts in channel expression and targeting. We here do not investigate the role of the extended helix C-D linker in neurons, and such experiments would be interesting in terms of nervous function.

How do the extra helix C-D linker residues affect channel surface expression? To address this question, we performed confocal imaging experiments between EYFP-tagged channels and an ER marker, pDsRED2-ER. Co-localization experiments suggest that the extra C-D linker residues in KCNQ3 are involved in the ER retention of channels. Consistent with this, the fraction of KCNQ3 (Δ16) channels trapped in the ER was lower compared to WT KCNQ3. Conversely, introduction of the same residues into the helix C-D linker of KCNQ4 led to a higher retention of KCNQ4 channels in the ER, consistent with the 16 extra residues constituting a novel ER retention signal. In the superfamily of Kv channels, several types of ER retention signals have been identified. These signals are mostly di- and tri-basic motifs such as di-arginine (RxR) or di-lysine (KKxx) where x represents any amino acid. Thus, Kv1.3 channels have a di-lysine motif (KKxx) in the C-terminus and a di-arginine motif (RXR) in the N-terminus [36]. A similar di-basic motif has been described in the N-terminus of Kv4.2 [37]. Unlike Kv1.3 and Kv4.2, the ER retention motif identified in Kv1.1 is not consisted of basic residues, but of four different residues (A352, E353, S369 and Y379), which are located within the turret-pore region [38–40].

For the family of KCNQ channels, only one ER retention signal has been clearly identified, and that in KCNQ1. As described in other Kv channels, this motif consists of basic residues (RxR) located in the S2-S3 linker of KCNQ1 [41]. In other KCNQ channels, none of the ER retention motifs described above have been found. However, several mutations in KCNQ1 leading to a “long QT syndrome” of cardiac arrhythmias (Y111C, L114P, and P117L) or Benign Familial Neonatal Seizures (R353G and L339R) mutations in KCNQ2 reduced channel expression via effects on trafficking of the channels [4,42,43]. All these mutations have been shown to increase the fraction of channels retained in ER, suggesting that Y111, L114, and P117 in KCNQ1, and also L339 and R353 in KCNQ2 may constitute, or be part of, an ER retention motif. Further experiments are required to confirm this hypothesis. In this study, we identified a possible new ER retention motif (SAFTFPSQQSPRNEPY) in the C-D helix linker within the distal C-terminus. Unlike other Kv channels, the motif describes here does not contain any well-established trafficking signals such as di- or tri-basic motifs. However, this motif contains several residues (proline, arginine, tyrosine, and serine) which may play a critical role in the retention of KCNQ3 in the ER as has been described in other Kv channels.

In this paper, we suggest that a putative novel ER retention motif within the helix C-D linker of KCNQ3 subunits partially contributes to differential channel surface expression, a signal that might be obscured in KCNQ2/3 heteromers. Thus, CHO cells expressing the KCNQ3 (Δ16) mutant with WT KCNQ2 subunits generated similar current amplitudes to co-expression of WT KCNQ2 + WT KCNQ3. Moreover, co-expression of mutant KCNQ2 (+16) with KCNQ3 (Δ16) subunits did not affect WT KCNQ2+3 current amplitudes, suggesting that such mutant channels can still co-assemble and traffic to the plasma membrane. Several studies have shown that heteromerization of other channels promotes channel trafficking by overcoming an ER retention signal. Thus, hERG 1b homomers, which possess an ER retention signal in the N-termini, generate very small currents [44]; whereas hERG 1b co-expressed with hERG 1a subunits produce hERG 1a/1b heteromers yielding robust currents [44–46]. Moreover, Phartiyal and co-workers demonstrated that hERG 1b protein exited the ER only when it is co-expressed with hERG 1a [47]. Similarly, in heart and brain, GIRK1 or GIRK4 expressed as homomers result in only minute currents, whereas co-expression of GIRK1+4 yield currents orders of magnitude larger [48,49]. Indeed, GIRK4 with the single S143F mutation express as homomers yielding very robust currents, a mutant GIRK subunit commonly used in ion channel studies, and referred to as GIRK4*, much like the single-mutant KCNQ3^T^ channels described in this work [48]. It will be interesting to further probe the parallels between mechanisms governing functional expression of GIRK and KCNQ channels. That both of these channel subtypes have open probabilities that are famously regulated by PIP_2_ makes such a possible analogy especially interesting.

As discussed above, considerable evidence suggests that the main difference in the profoundly divergent macroscopic current amplitudes, i.e., functional channel expression, between WT KCNQ3, KCNQ3 A315T,S or KCNQ4 arises not from divergent channel assembly, but rather from an intrinsically unstable pore of WT KCNQ3, compared to the others, which is stabilized by the presence of a hydrophilic residue at the internal pore, instead of the alanine at position 315 in WT KCNQ3 [6,16], or assembly with KCNQ2 subunits. This unstable pore or “dormant channel” hypothesis has strong analogy to the unstable pore in various KcsA mutants [50–53] or to a variety of K^+^ channels malfunctioning in the absence of K^+^ [54–57]. Taken together, we conclude that multiple processes act in concert to govern functional activity of M-type K^+^ channels, with mechanisms localizing both to the pore, to the distal carboxy terminus, and possibly also to the amino terminus.

In summary, we identified 16 residues in the helix C-D linker of KCNQ3 that may constitute an ER retention motif that contributes to smaller current amplitudes compared to other KCNQ channels. Given that these 16 residues are absent in other KCNQ channels, we hypothesize that the differential channel expression among KCNQ channels is partially due to this divergent helix C-D linker. Finally, the higher current amplitude of KCNQ2/3 heteromers is likely partially caused by masking this novel ER retention motif located in the helix C-D linker of KCNQ3.
